# Neural Activity Correlates With Behavior Effects of Anti-Seizure Drugs Efficacy Using the Zebrafish Pentylenetetrazol Seizure Model

**DOI:** 10.3389/fphar.2022.836573

**Published:** 2022-04-12

**Authors:** Patrick C. Milder, Agnes S. Zybura, Theodore R. Cummins, James A. Marrs

**Affiliations:** Department of Biology, Indiana University Purdue University Indianapolis, Indianapolis, IN, United States

**Keywords:** epilepsy, zebrafish, pentylenetetrazol (PTZ), behavior models, anti-seizure drugs

## Abstract

Approximately 30% of patients with epilepsy do not achieve adequate seizure control through current anti-seizure drugs and treatment methods. Therefore, a critical need exists to efficiently screen anti-seizure drugs to enhance our ability to tailor treatment protocols and improve patient outcomes. The zebrafish pentylenetetrazol (PTZ) seizure model has become an increasingly popular screening paradigm for novel anti-seizure compounds. However, previous research using this model was variable due to differing experimental methods. Here, we present a method that was optimized to improve reliability and reproducibility in our laboratory using this PTZ model to develop a more robust screening of anti-seizure drugs comparing behavior and neural activity. Our behavior assay, spanning 90 min using 10 mM PTZ on 7 days post fertilization zebrafish, provides a broad window to observe anti-seizure drug efficacy. To compare our method with previously published data, we tested carbamazepine, lamotrigine, and topiramate, which have been tested in previous PTZ zebrafish assays. In addition, we assessed the candidate anti-seizure compound GS967, which has not been previously tested in the zebrafish seizure model. We examined the efficacy of anti-seizure drugs by acute administration concurrent with PTZ application and by pretreatment prior to exposure with PTZ. Pretreatment permitted us to examine potential neuroprotection and determine whether treatment time affects anti-seizure drugs’ responses. As independent validation of anti-seizure drugs’ effects, we evaluated whether the anti-seizure drug efficacy in the behavioral assay correlated with neural activity measurements, using electroencephalogram (EEG) and calcium signaling using GCaMP. There was no significant difference in the reduction of PTZ-induced seizure behavior activity between the pretreatment groups and acute treatment groups. Acute treatment with anti-seizure drugs in the EEG and GCaMP assays from 15 to 30 min post-anti-seizure drug exposure revealed consistent results between behavioral, EEG, and GCaMP assays for two of the three anti-seizure drugs. Lamotrigine only reduced neural activity (EEG and GCaMP assays). Carbamazepine, topiramate, and GS967 reduced activity in all three assays. The findings show that EEG and GCaMP assays largely correlate with the behavior findings, helping us connect physiological and behavior responses to anti-seizure drug and better assess anti-seizure drug efficacy.

## Introduction

Epilepsy is a disease classified by the recurrent state of seizures due to an imbalance between neuronal inhibition and excitation (Scharfman, 2007). Physicians manage individual patients with epilepsy, often by trying different medications to effectively control their seizures. However, using currently available treatments, at least 30% percent are unable to achieve adequate seizure control (Romanelli et al., 2012). There is a critical need to screen current and novel anti-seizure drugs to more effectively aid those individuals who are treatment refractory.

The zebrafish pentylenetetrazol (PTZ) seizure behavioral model has been employed to test anti-seizure drugs using a variety of methods, producing divergent results across the field because no standard protocol currently exists (Baraban et al., 2005; Gupta et al., 2014; Afrikanova et al., 2013; Kundap et al., 2017). For example, zebrafish adults were used to evaluate anti-seizure drugs on a PTZ treated behavior paradigm (Gupta et al., 2014; Kundap et al., 2017). Also, different concentrations of PTZ treatments using larvae are reported: 15–20 mM (Afrikanova et al., 2013) and 2.5–15 mM (Baraban et al., 2005). There are also various lengths of observation windows, and administration routes of anti-seizure drugs being used. Afrikanova et al. (2013) pretreated 7 days post fertilization (dpf) zebrafish with anti-seizure drugs for 18 h before PTZ was added, and the fish were given 5 min to habituate to a dark chamber before monitoring and quantifying movement for 30 min using a ZebraBox™. Baraban et al. (2005) took 2 min recordings of seven dpf zebrafish in control medium using a CCD camera and locomotion tracking software, comparing this to fish that were placed in 2.5–15 mM PTZ solution for 10 min. Again, 2 min recordings of movements were quantified. They also evaluated seizure type and percentage of fish affected.

The experimental approach was standardized and optimized in our laboratory to evaluate seizure activity in zebrafish, including behavioral, EEG, and GCaMP assays, producing reliable, and comparable results and to test a candidate anti-seizure compound. The zebrafish PTZ model has construct validity by inducing seizures, and it models seizure events in humans providing face validity. Previously tested anti-seizure drugs that work in humans also reduce seizures in the zebrafish PTZ model, indicating potential predictive validity. This study optimized the zebrafish PTZ model of seizure by examining the impact of varied PTZ concentrations and the duration of its effects. We tested carbamazepine, lamotrigine, topiramate, and the candidate anti-seizure compound, GS967 (a persistent sodium channel modulator; Anderson et al., 2014; Baker et al., 2018). Using longer assay time (90 min) and a concentration of 10 mM PTZ allowed for a broader view of anti-seizure drug efficacy. We tested carbamazepine, lamotrigine, topiramate which have been tested in previous PTZ zebrafish assays (Baraban et al., 2005; Gupta et al., 2014; Afrikanova et al., 2013). We also evaluated the candidate anti-seizure compound GS967 in our PTZ model, which has been used in mouse models and shown to be specifically effective for SCN8A (Baker et al., 2018) and SCN2A epilepsy (Anderson et al., 2014). We used our optimized assay to then test two hypotheses: 1) the efficacy of anti-seizure drugs may depend on whether anti-seizure drugs are administered acutely with PTZ or whether with pretreatment of the anti-seizure drugs before PTZ exposure; and 2) the efficacy of anti-seizure drug variability is consistent using different assays, like behavior, EEG, and GCaMP. Pretreatment versus acute application permitted us to examine whether anti-seizure drug/candidate pretreatment protects against PTZ induced seizures or whether only acute administration is needed. The outcomes of our experiments provide a consistent and effective model that will facilitate comparisons of various drugs.

Comparing different anti-seizure drugs using one reliable behavioral assay allowed us to resolve contradictory behavioral assay results in the literature. Observing behavior with the assistance of Viewpoint ZebraBox™ technology allowed for a consistent and high throughput assessment of anti-seizure drug efficacy. EEG readings and GCaMP imaging provided an independent evaluation of seizure activity for the behavioral assay. Using these zebrafish assays, rapid and robust comparison were made between known anti-seizure drugs and a candidate anti-seizure compound.

## Materials and Methods

### Zebrafish Husbandry

The Indiana University Policy on Animal Care and Use guidelines were followed, and all experiments and procedures were approved by the IUPUI School of Science Institutional Animal Care and Use Committee. Zebrafish (*Danio rerio*) AB strain were raised and maintained under standard laboratory conditions (Westerfield, 2007). Fertilized eggs were collected from mating chambers and rinsed with embryo medium (EM). Zebrafish larva and embryos were maintained at 28.5°C, on a 14/10 h light/dark cycle under standard conditions.

### Drug Administration

At six dpf, zebrafish were moved to individual wells of a 96-well plate and placed in 300 μL EM. Two different methods were used to administer anti-seizure drugs: pretreatment and acute. The pretreated groups were placed in 150 μl EM along with 150 μl at 2x of the final concentration of the respective anti-seizure drugs 24 h before the behavioral assay was performed. Thus seven dpf was the development stage used for EEG, GCaMP, and behavioral testing. Ten to 12 larvae were used per treatment parameter, and five to eight 96-well plates were used per experiment. All groups were tested together on each plate to control for potential differences in per plate variation. All larvae were raised in the same embryo medium and in the same Petri plate to control for variation in incubation conditions. On day 7, larvae were allowed to habituate for 30 min in the light at room temperature. After pre-incubation, 300 µl of embryo medium or 300 µl of a 2x solution for the acute group was added to obtain a final concentration of 1x for all treatment groups. This method was used for all larvae in all experiments.

Three anti-seizure drugs, carbamazepine (CBZ), lamotrigine (LTG), topiramate (TPR), and one candidate anti-seizure compound, GS967 were tested in our assay. Anti-seizure drugs were tested at several concentrations using previous studies as a guide. The candidate compound, GS967, has not been tested in zebrafish and required more testing at several concentrations (data not shown). Optimal concentrations were reported as follows. CBZ and LTG were both used at a final concentration of 100 µM. TPR was used at a final concentration of 200 µM in line with previous research (Bradford, 1995; Afrikanova et al., 2013; Gupta et al., 2014). As GS967 had not been used previously in zebrafish PTZ behavioral assays, a dose response test was performed, and 0.05 µM GS967 was found to be the most effective concentration at reducing seizure activity without anesthetizing the fish (i.e., high enough to reduce seizures, low enough that the control fish still move). These concentrations remained consistent throughout all assays. The anti-seizure drugs were evaluated based on duration and acute vs pretreatment. Only acute treatment of anti-seizure drugs was used in the EEG and GCaMP Assays.

### Movement Tracking System

After adding PTZ, larvae were immediately moved to an automated tracking device, the ZebraBox™ apparatus. The large movement count was then quantified using ZebraLab™ software. Large count activity is defined as the number of movement events over a speed of 8 mm/s. Integration periods were grouped into 15 min intervals. Locomotion was tracked and measured over a total 90 min assay. This longer period of tracking differs from previous studies that used shorter periods. The ZebraBox™ collects data on fish movement, eliminating subjectivity in the observation of seizures to control for experimental bias.

### GCaMP Assay

A seven dpf larva was embedded in 1% low-melting-point agarose. The plate containing the embedded larva had 2 ml of EM added to it. The larvae were allowed to habituate in embryo medium for 10 min. Once the initial 10 min of habituation had concluded, a Z-stack image was taken using a confocal microscope using a ×10 objective (Zeiss LSM 700). The maximum projection feature was used to create a single image of the zebrafish larva’s baseline synaptic activity in EM. The GCaMP assay treatments were not blinded. An equal volume of 2x solution (DMSO, anti-seizure drug, PTZ or PTZ + anti-seizure drug) was then added to each plate and the larva was given 15 min to incubate in the testing solution. After 15 min a second Z-stack image was taken and once again the 21 slices were used to create a single image *via* maximum projection. All settings remained the same between both Z-stack sessions. The GCaMP assay used ImageJ for objective measurement of fluorescence.

### EEG Assay

Each 7 dpf larva was then embedded in 1% low-melting-point agarose. The plate containing the embedded larva had 2 ml of EM added to it. A glass electrode filled with 2 M NaCl was placed into the optic tectum and recordings were performed in current clamp mode, low-pass filtered at 1 kHz, high-pass filtered at 0.1 Hz, digital gain 10, sampling interval 10 µs (EPC10, Heka Electronic). The larvae were allowed to habituate with electrode in place for 10 min. Once the initial 10 min of habituation was complete, a 10 min baseline reading occurred. The EEG treatments were not blinded. An equal volume of 2x solution (DMSO, anti-seizure drug, PTZ or PTZ + anti-seizure drug) was then added to each plate. The recordings started each time exactly 5 min after removal of the larva from proconvulsant solution and were continued for 10 min. Thus, EEG recordings were performed consistently from minutes 20 through 30 following exposure to PTZ. Recordings from eight larvae were taken per experimental condition. Seizure activity was analyzed according to the amount of spiking paroxysmal events or local field potentials (LFP). The EEG assay uses a set level of voltage (2 mV) to standardize the evaluation of potential seizure activity. This threshold is approximately 5 times the noise level and allowed detection of major events in the recordings.

### Statistical Analysis

ARRIVE guidelines 2.0 were used in our animal experiments for reproducibility and rigor assurance. Sample sizes of larvae were determined using pilot experiments rather than power analysis to determine numbers needed. This approach was pursued because the ZebraBox™ apparatus has a variable number of wells in the plates that show excessive background. Thus, we have variable numbers of larvae per experiment. All larvae were included that did not show background in the measurements.

### Behavior

Locomotor behavior data from replicate tracking run time points were subsequently averaged and analyzed by two-way ANOVA. Tukey’s post-hoc comparisons were appropriate. The total large counts of each treatment group within 90 min were compared using one-way ANOVA followed by a Tukey’s post hoc test where appropriate (GraphPad Prism software version 8.2). Embryo medium was used as the control group for untreated, dimethyl sulfoxide (DMSO) was used as the vehicle control, and 10 mM PTZ groups were used to represent the untreated epileptic condition.

### GCaMP

In order to assess calcium signaling, a line of Tg (elavl3:GCaMP6s) zebrafish were obtained from the zebrafish resource center (zfin.org). The fish were heterozygous for the transgenic gene upon arrival and were backcrossed until a homozygous line was created and kept. We used these homozygous Tg (elavl3:GCaMP6s) zebrafish for our calcium signaling experiments. Fluorescence of the midbrain was measured using ImageJ. The initial image was measured and normalized to 1. The treated image fluorescence was divided by the initial image fluorescence and was recorded as a normalized value compared to the initial embryo medium fluorescence. We followed this procedure for all treatments. Normalizing the fluorescence based on the same fish allowed us to rule out noise based on differences in fluorescence scores between different larvae. The normalized fluorescence scores were grouped by solution and were compared using one-way ANOVA followed by a Tukey’s post hoc test (GraphPad Prism software version 8.2). Embryo medium was used as the control group for untreated, dimethyl sulfoxide (DMSO) was used as the vehicle control, embryo medium-15 (EM-15), which was used as the control group for the 15 min time difference in between Z-stacks, and 10 mM PTZ groups were used to represent the untreated epileptic condition.

### EEG Assay

We took the total number of spiking paroxysmal events in the initial baseline recording. We then divided the total number of spiking paroxysmal events during baseline for individual larvae by the total number of spiking paroxysmal events during treatment for the same larvae to eliminate variability between different larvae. All spikes of 2 mV or higher were counted and totaled to compare the EEG spikes before and after administration of treatments. The initial baseline paroxysmal events count was normalized to one, which was divided by the paroxysmal events after treatment. Scores were grouped by solution and were compared using one-way ANOVA followed by a Tukey’s post hoc test (GraphPad Prism software version 8.2). Embryo medium was used as the control group for untreated, dimethyl sulfoxide (DMSO) was used as the vehicle control, and embryo medium-15 (EM-15), which was used as the control group for the 15 min time difference in between recordings and 10 mM PTZ groups were used to represent the untreated epileptic condition.

## Results

### Anti-Seizure Drugs Effects on PTZ-Induced Seizure Behavioral Activity of Zebrafish 7 dpf Larvae: Assay Description

**FIGURE 1:**
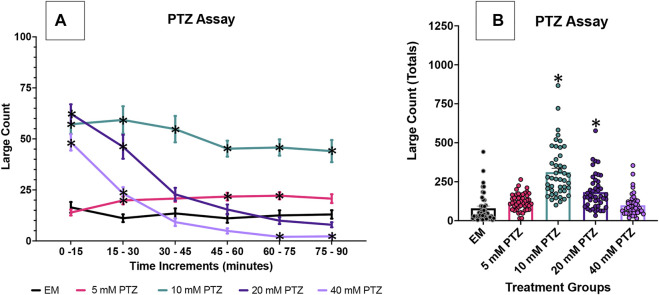
Behavioral profile of zebrafish larvae exposed to PTZ. The following graphs displays the average large count (*y*-axis) of larvae. **(A)** The average larval large count is depicted per 15 min interval (*x*-axis) of the tracking session. Zebrafish larvae were tested PTZ concentrations at 5 mM, 10 mM, 20 mM, and 40 mM (n = 70 fish/solution/concentration). Embryo medium (EM) was used as the control. Significance indicated by *p < 0.05 PTZ vs. EM, two-way ANOVA, Tukey’s post-hoc. **(B)** The total large count (*y*-axis) over 90 min is displayed. The respective treatment groups (*x*-axis). Analyses (one-way ANOVA, Tukey’s post-hoc) are marked * (*p* < 0.05) to indicate significant differences to embryo medium (EM).

We first investigated the effects of several PTZ concentrations on the zebrafish behavior profile over an extended 90 min time duration. Concentrations of 10 mM PTZ, 20 mM PTZ and 40 mM PTZ produced robust seizure activity. With 5 mM PTZ, an increased large count was observed but was inconsistent between replicates. 10 mM PTZ produced seizure activity that was more consistent than other concentrations over the course of the 90 min assay in repeated trials. In contrast, 20 and 40 mM PTZ significantly increased large (>8 mm/s) movement counts at the beginning of the assay (Figure 1). However, the seizure-like behavior subsided substantially over the course of the 90 min assay.

Given the consistent increases in seizure activity over 90 min seen in 7 dpf zebrafish larvae exposure, 10 mM PTZ was used as the optimal concentration for the experiments exploring the effectiveness of three distinct anti-seizure drugs and GS967, a candidate anti-seizure compound, on reducing PTZ-induced seizure activity (Figure 1; Supplementary Tables S1‐S7).

**FIGURE 2:**
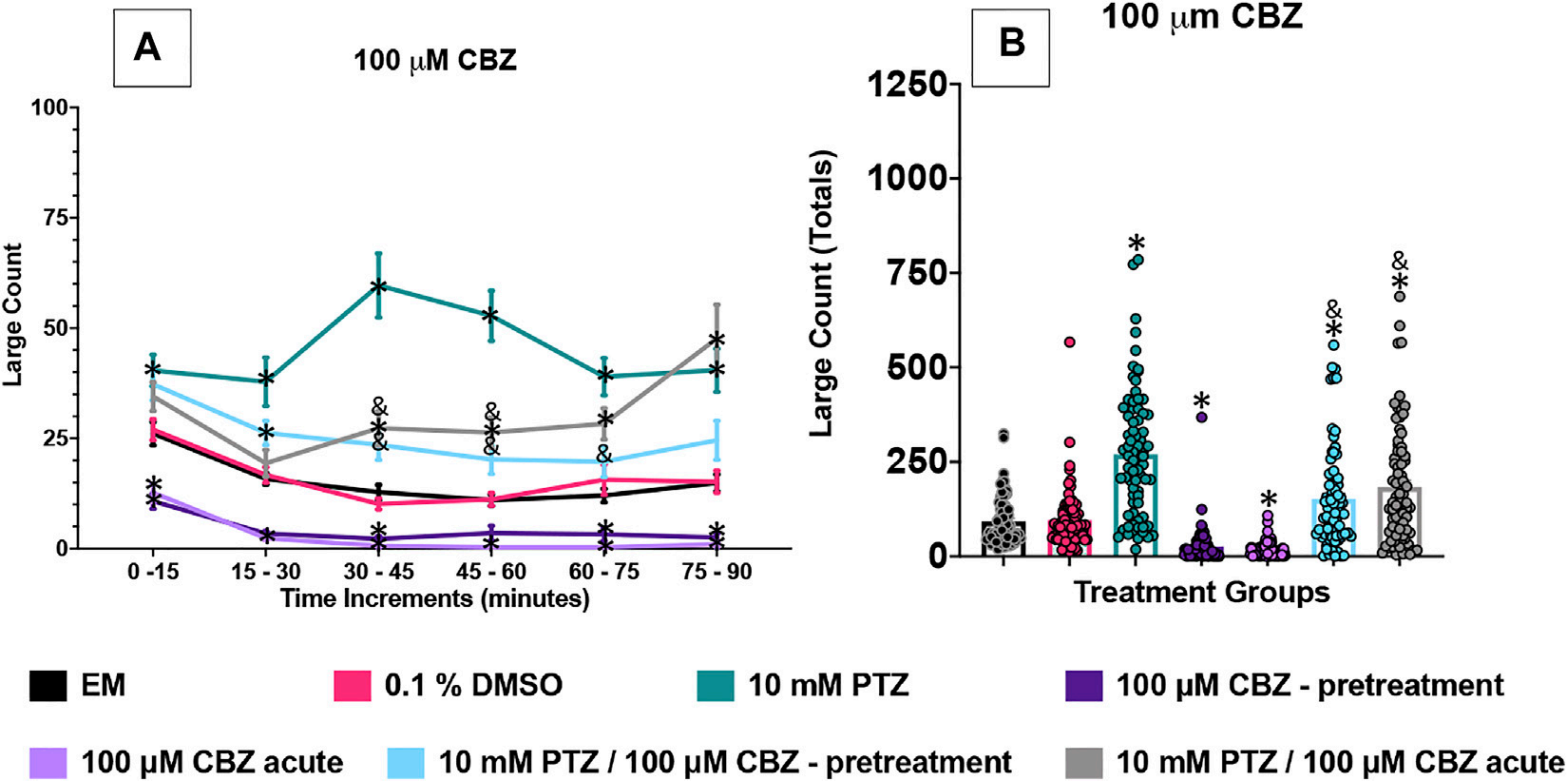
Behavioral profile of zebrafish larvae exposed to carbamazepine **(A)** displays the average large count (*y*-axis) of larvae. The average larval large count is depicted per 15 min interval (*x*-axis) of the tracking session. The respective treatment groups are listed on the *x*-axis. Analyses (two-way ANOVA, Tukey’s post-hoc) are marked * (*p* < 0.05) to indicate significant differences to embryo medium (EM). Standard error of the means is shown. Analyses (two-way ANOVA, Tukey’s post-hoc) are marked and (*p* < 0.05) to indicate significant differences to 10 mM PTZ. **(B)** The total large count (*y*-axis) over 90 min is displayed. The respective treatment groups (*x*-axis). Analyses (one-way ANOVA, Tukey’s post-hoc) are marked * (*p* < 0.05) to indicate significant differences to embryo medium (EM). Standard error of the means is shown. Analyses (one-way ANOVA, Tukey’s post-hoc) are marked & (*p* < 0.05) to indicate significant differences to 10 mM PTZ. (*n* = 76 fish/treatment group).

**FIGURE 3:**
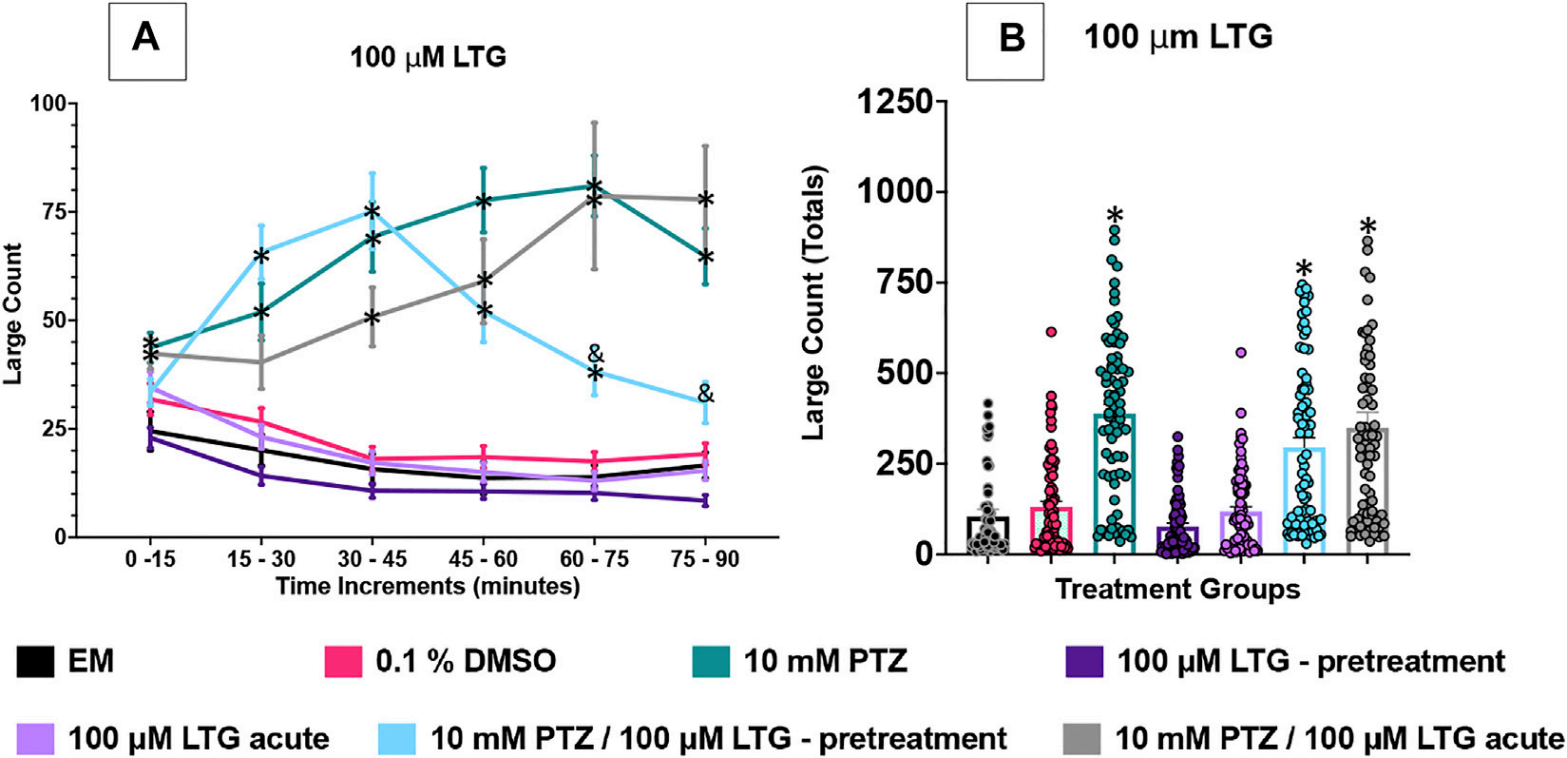
Behavioral profile of zebrafish larvae exposed to lamotrigine. **(A)** The average large count (*y*-axis) of larvae is depicted per 15 min interval (*x*-axis) of the tracking session. The respective treatment groups are listed on the *x*-axis. Analyses (two-way ANOVA, Tukey’s post-hoc) are marked * (*p* < 0.05) to indicate significant differences to embryo medium (EM). Standard error of the means is shown. Analyses (two-way ANOVA, Tukey’s post-hoc) are marked & (*p* < 0.05) to indicate significant differences to 10 mM PTZ. **(B)** The total large count (*y*-axis) over 90 min is displayed. The respective treatment groups (*x*-axis). Analyses (one-way ANOVA, Tukey’s post-hoc) are marked * (*p* < 0.05) to indicate significant differences to embryo medium (EM). Standard error of the means is shown. Analyses (one-way ANOVA, Tukey’s post-hoc) are marked & (*p* < 0.05) to indicate significant differences to 10 mM PTZ. (*n* = 76 fish/treatment group).

**FIGURE 4:**
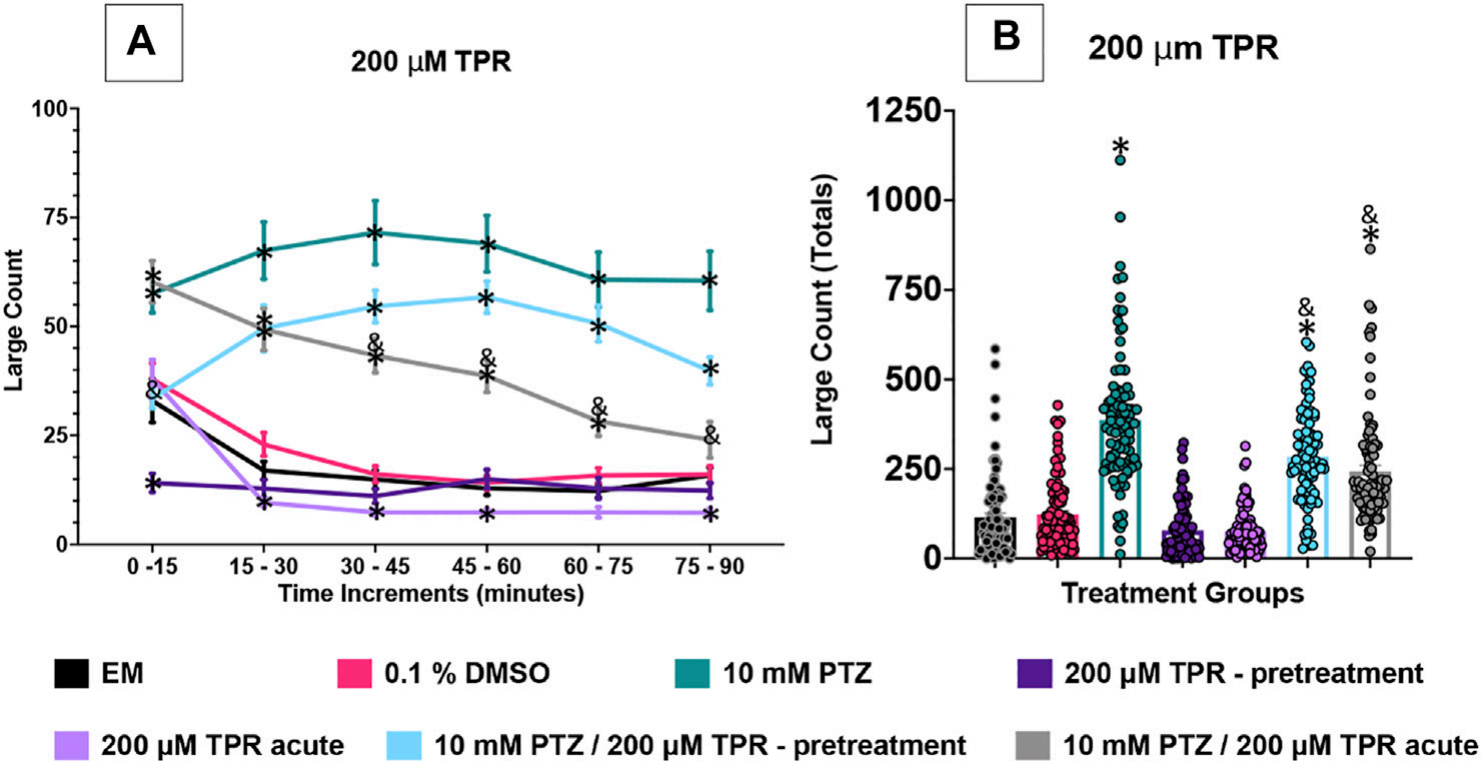
Behavioral profile of zebrafish larvae exposed to topiramate. **(A)** The average large count (*y*-axis) of larvae is depicted per 15 min interval (*x*-axis) of the tracking session. The respective treatment groups (*x*-axis). Analyses (two-way ANOVA, Tukey’s post-hoc) are marked * (*p* < 0.05) to indicate significant differences to embryo medium (EM). Standard error of the means is shown. Analyses (two-way ANOVA, Tukey’s post-hoc) are marked & (*p* < 0.05) to indicate significant differences to 10 mM PTZ. **(B)** The total large count (*y*-axis) over 90 min is displayed. The respective treatment groups (*x*-axis). Analyses (one-way ANOVA, Tukey’s post-hoc) are marked * (*p* < 0.05) to indicate significant differences to embryo medium (EM). Standard error of the means is shown. Analyses (one-way ANOVA, Tukey’s post-hoc) are marked & (*p* < 0.05) to indicate significant differences to 10 mM PTZ. (*n* = 91 fish/treatment group).

**FIGURE 5:**
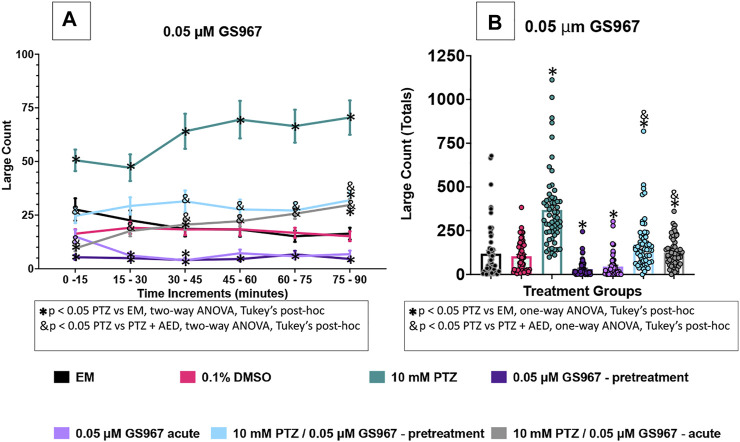
Behavioral profile of zebrafish larvae exposed to GS967. **(A)** The average large count (*y*-axis) of larvae. The average larval large count is depicted per 15 min interval (*x*-axis) of the tracking session. The respective treatment groups (*x*-axis). Analyses (two-way ANOVA, Tukey’s post-hoc) are marked * (*p* < 0.05) to indicate significant differences to embryo medium (EM). Standard error of the means is shown. Analyses (two-way ANOVA, Tukey’s post-hoc) are marked & (*p* < 0.05) to indicate significant differences to 10 mM PTZ. **(B)** The total large count (*y*-axis) over 90 min is displayed. The respective treatment groups (*x*-axis). Analyses (one-way ANOVA, Tukey’s post-hoc) are marked * (*p* < 0.05) to indicate significant differences to embryo medium (EM). Standard error of the means is shown. Analyses (one-way ANOVA, Tukey’s post-hoc) are marked & (*p* < 0.05) to indicate significant differences to 10 mM PTZ. (*n* = 60 fish/treatment group).

Figures 2–5 show the results of the various anti-seizure drugs and anti-seizure compound assays performed over an extended 90 min duration in independent assays. There were seven treatment groups in each assay. The groups were as follows: embryo medium; 0.1% DMSO: 10 mM PTZ; anti-seizure drug alone (pretreated); anti-seizure drug alone (acute); 10 mM PTZ + anti-seizure drug (pretreated); and 10 mM PTZ + anti-seizure drug (acute). Significance indicated vs. EM by * and vs. PTZ alone by &.

### CBZ Effects on Seizure Behavioral Activity in 7 dpf Larvae

In this set of experiments, the effect of 100 µM CBZ was examined on the zebrafish activity. Compared to EM exposed zebrafish, acute exposure to CBZ alone (no PTZ exposure) induced a significant decrease in large movement counts throughout all six time periods (0–90 min). A decrease in activity was observed at four of the six assay time periods: 0–15 min, 30–45 min, 60–75 min, and–90 min. By contrast, the vehicle, DMSO, did not impact zebrafish behavior. Zebrafish exposed to PTZ (10 mM) exhibited a significant increase in large movement counts (compared to EM) across six time periods (0–90 min).

A significant decrease in large movement counts was observed during three time periods (30–45 min, 45–60 min and 60–75 min) when pretreatment with CBZ was followed by PTZ, and during two time periods (30–45 min and 45–60 min) for the acute CBZ group where CBZ and PTZ exposures were compared (Figure 2A).

To further compare these data, we summed the large count over the entire 90 min assay for the various treatment groups (Figure 2B). The DMSO and CBZ-pretreatment alone groups had similar large count totals compared to EM. Acute CBZ treatment alone decreased total large count movement (Figure 2B) compared to EM. PTZ significantly increased large count movement compared to EM. CBZ, both acute and pretreated reduced PTZ induced seizure activity over multiple time periods (Figure 2A) and in total large count (Figure 2B).

### LTG Effects on Seizure Behavioral Activity in 7 dpf Larvae

The effect of 100 µM LTG was examined on the zebrafish activity. Compared to EM exposed zebrafish, DMSO, the vehicle, did not significantly impact zebrafish behavior and neither did the addition of LTG alone regardless of administration method (acute or pretreatment). In this set of experiments, zebrafish exposed to PTZ (10 mM) exhibited a significant increase in large movement counts (compared to EM) across all six time periods (0–90 min). PTZ with acute LTG administration did not significantly reduce seizure activity at any individual time point However, PTZ with larvae pretreated with LTG did experience significantly reduced large movement counts for the last 30 min of the assay compared to PTZ alone. To further compare these data, we summed the large count over the entire 90 min assay for the seven treatment groups shown in Figure 3B. The DMSO, LTG-acute alone, and LTG-pretreatment alone groups had similar large count totals compared to EM. PTZ, PTZ + LTG (both acute and pretreated) significantly increased large count movement compared to EM. LTG, both acute and pretreated, did not significantly reduce PTZ induced seizure activity in total large count (Figure 3B).

### TPR Effects on Seizure Behavioral Activity in 7 dpf Larvae

The effect of 200 µM TPR was examined on the zebrafish activity. Compared to EM exposed zebrafish, acute exposure to TPR alone induced a significant decrease in large movement counts in four time periods (15–30 min, 30–45 min, 45–60 min, and 75–90 min). With TPR pre-treatment alone, a decrease in large movement counts was observed at two time periods (0–15 min and 15–30 min). DMSO did not impact zebrafish behavior. The zebrafish exposed to PTZ (10 mM) exhibited a significant increase in large movement counts (compared to EM) across all six time periods (0–90 min).

The large movement counts were summed over the entire 90 min assay for the various TPR treatment groups (Figure 4B). The DMSO, and both TPR groups had similar large count totals compared to EM. The total large movement counts for both the TPR pretreatment + PTZ group and the acute TPR + PTZ group were significantly lower than the PTZ group total large counts.

### GS967 Effects on Seizure Behavioral Activity in 7 dpf Larvae

In this set of experiments the effect of 0.05 µM GS967 was examined on the zebrafish activity. Compared to EM exposed zebrafish, acute exposure to GS967 alone induced a significant decrease in large movement counts throughout three time periods (15–30 min, 30–45 min, and 60–75 min). With GS967 pre-treatment, a decrease in activity was observed at five of the six time periods (0–60 min, 75–90 min). By contrast, the vehicle, DMSO did not impact zebrafish behavior. Zebrafish exposed to PTZ (10 mM) exhibited a significant increase in large movement counts (compared to EM) across six time periods (0–90 min).

A significant decrease in large movement counts was observed during five time periods (0–15 min, 30–45 min, 60–75 min and 75–90 min) when pretreatment with GS967 was followed by PTZ, and during six time periods (0–90 min) for the acute GS967 group when GS967 and PTZ exposures were compared to PTZ alone (Figure 5A).

To further compare these data, we summed the large count over the entire 90 min assay for the various treatment groups (Figure 5B). The DMSO group had similar large count totals compared to EM. GS967 treatments decreased total large count movement (Figure 5B) compared to EM. PTZ significantly increased large count movement compared to EM and GS967 with PTZ groups. Acute and pretreated GS967 both reduced PTZ induced seizure activity over multiple time periods (Figure 5A) and in total large count (Figure 5B). Interestingly, GS967 with PTZ groups had similar large count movements to EM.

### PTZ and Anti-Seizure Drug Effects on Calcium Signaling Activity in 7 dpf Larvae

Transgenic Tg (elavl3:GCaMP6s) zebrafish have been used to monitor PTZ induced seizures (Turrini et al., 2017). We were able to observe and measure the difference in fluorescence between baseline activity in embryo medium, the increased fluorescence seen once zebrafish were allowed to habituate in a 10 mM PTZ solution, and the difference between PTZ fluorescence and PTZ + anti-seizure drug. The midbrain was chosen as the region of interest to measure so we would be able to pair it with the EEG data in which the probe is placed in the optic tectum, the largest midbrain structure (Baraban et al., 2007). We found that in the behavioral experiments there was not a significant difference between the pretreatment groups and acute treatment groups’ ability to reduce PTZ induced seizure activity for each of the anti-seizure drugs tested. Based on this result, we tested only acute treatment of the anti-seizure drug in the EEG and GCaMP assays using the observation window of 15 min post exposure to 30 min post exposure to see if the results matched the results observed in the behavioral assays.

**FIGURE 6:**
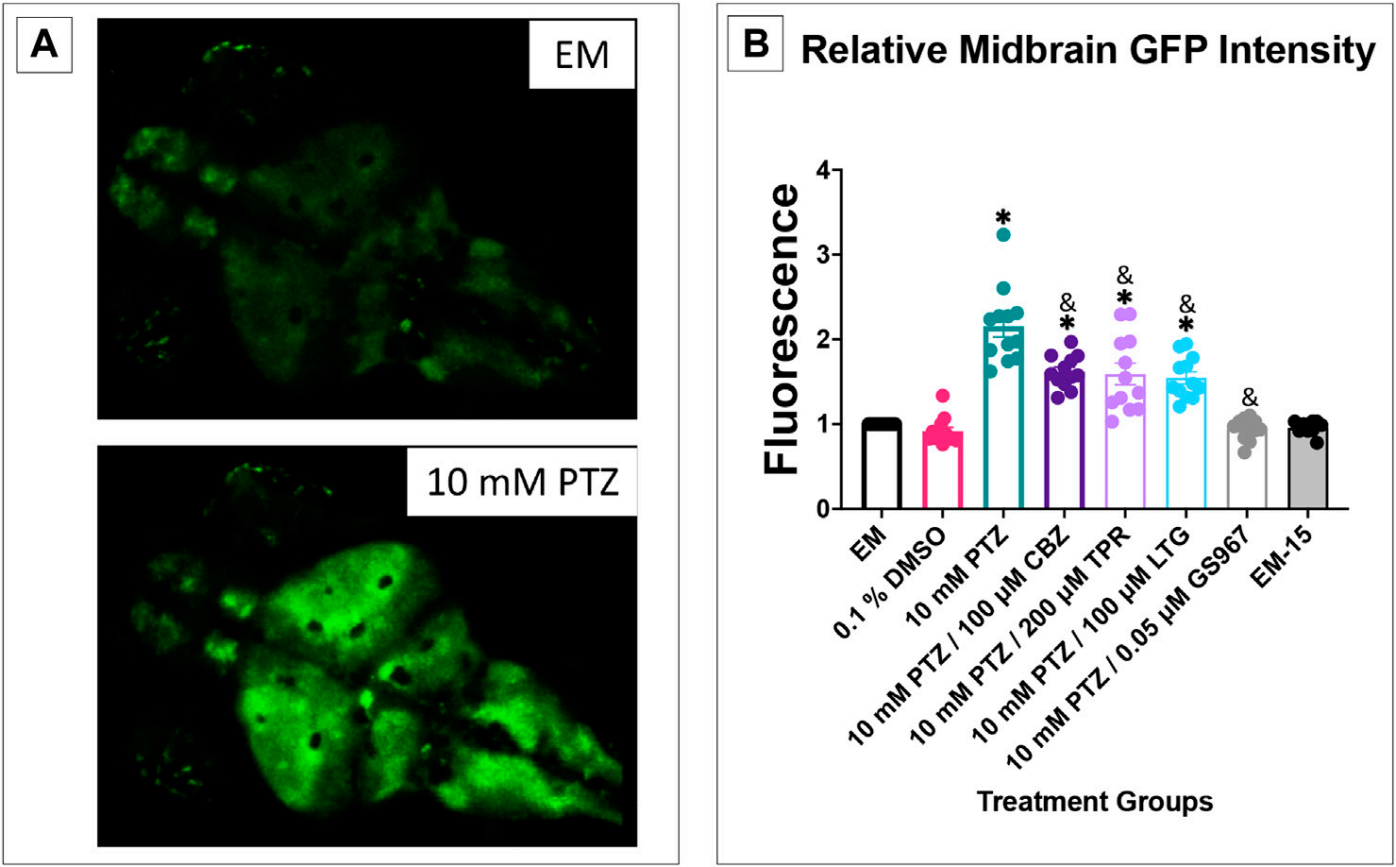
Calcium signaling activity in 7 dpf larvae. The fluorescence score is displayed on the *y*-axis. The respective treatment groups are shown on the *x*-axis. Analyses (one-way ANOVA, Tukey’s post-hoc) are marked * (*p* < 0.05) to indicate significant differences to embryo medium (EM). Standard error of the means is shown. Analyses (one-way ANOVA, Tukey’s post-hoc) are marked & (*p* < 0.05) to indicate significant differences to 10 mM PTZ. (*n* = 12 fish/treatment group).

It was observed during this experiment that DMSO, EM-15, and PTZ + GS967 all had similar fluorescence scores compared to EM (Figure 6). PTZ had significantly higher fluorescence than EM and all PTZ + anti-seizure drug groups. This result further correlates the ability of the four respective anti-seizure drugs/compound (CBZ, LTG, TPR, and GS967) ability to reduce seizure activity and corroborates the data shown in the behavioral assays.

### PTZ and Anti-Seizure Drug Effects on EEG Activity in 7 dpf Larvae

We observed and measured the difference in the number of LFP for baseline activity in embryo medium, once zebrafish were allowed to habituate in a 10 mM PTZ solution, and additionally the difference between PTZ and PTZ + anti-seizure drugs. EEG data was taken from the optic tectum providing consistency with calcium signaling data. Choosing the optic tectum allowed us to target a portion of the brain that could be consistently seen and probed similarly between all groups and larvae, thus, reducing variability between individual zebrafish.

**FIGURE 7:**
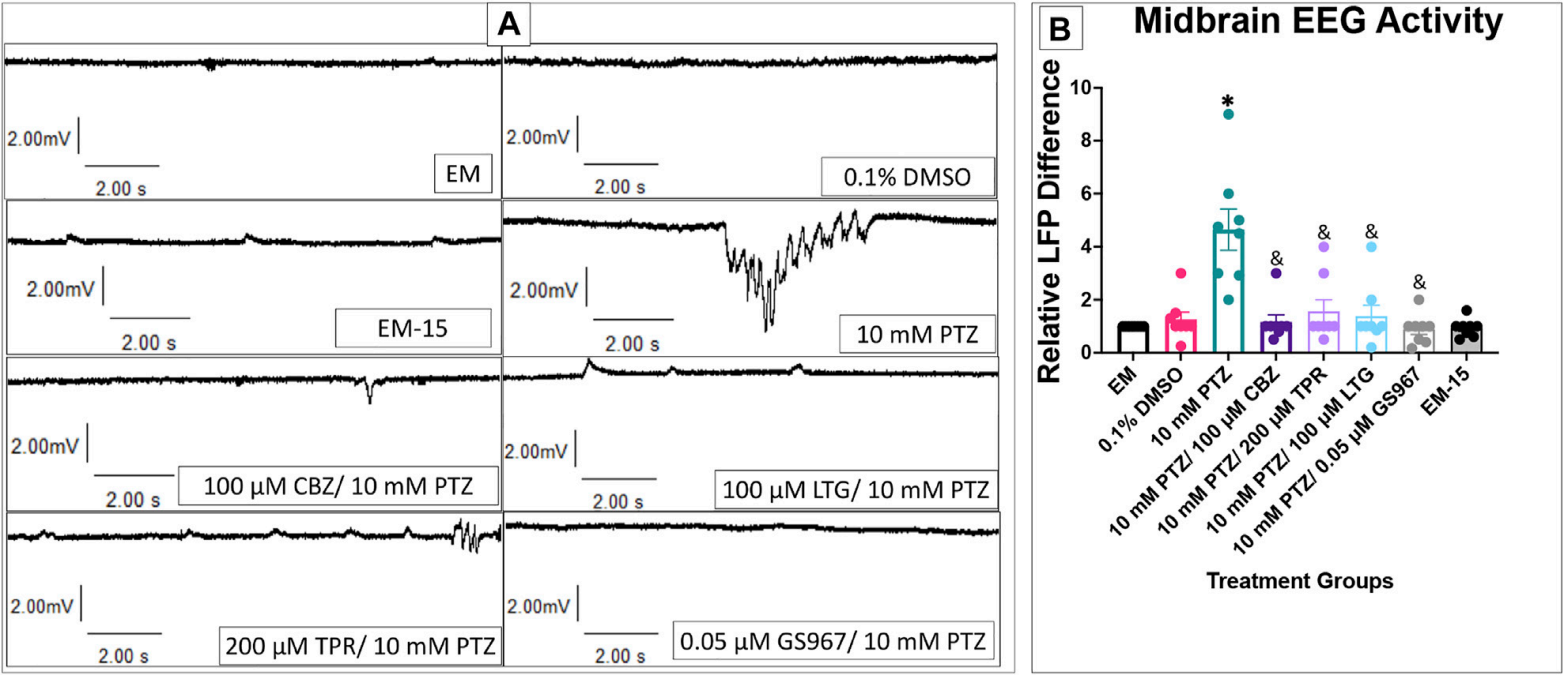
Local field potentials in 7 dpf larvae. **(A)** Representative tracings recorded in the presence of EM (top) and 10 mM PTZ (bottom) are shown. **(B)** The relative LFP difference is displayed on the *y*-axis. The respective treatment groups are shown on the *x*-axis. Analyses (one-way ANOVA, Tukey’s post-hoc) are marked * (*p* < 0.05) to indicate significant differences to embryo medium (EM). Standard error of the means is shown. Analyses (one-way ANOVA, Tukey’s post-hoc) are marked & (*p* < 0.05) to indicate significant differences to 10 mM PTZ. (*n* = 8 fish/treatment group).

DMSO, EM-15, and all PTZ + anti-seizure drug groups had similar LFP counts compared to EM (Figure 7). PTZ had significantly higher LFP than EM and all PTZ + anti-seizure drug groups. This result, in combination with the GCaMP and behavioral assays results, corroborates the reliability of the behavioral model in measuring an anti-seizure drugs effectiveness on reducing seizure activity.

## Discussion

Inconsistencies in methods evaluating anti-seizure drugs could make it difficult to determine the relative effects. Our findings evaluated PTZ and anti-seizure effects over a longer time period using a moderate PTZ concentration. Higher PTZ concentrations can produce synaptic fatigue, exhaustion, or death, producing a reduction in swimming behavior in later time increments. This variability could confound comparisons of anti-seizure drugs.

The optimized PTZ assay conditions were used to determine whether three clinically useful anti-seizure drugs and a candidate anti-seizure compound exhibited efficacy against 10 mM PTZ induced seizure like behavior when given acutely with PTZ or pretreated for 24 h with the anti-seizure drug before PTZ exposure. Large movement counts were compared in our assay, but other measures, including small movement counts, large distance travelled, and small distance travelled, also showed effects of the PTZ and anti-seizure compounds. The large movement measure showed the most sensitivity. Anti-seizure drugs were also tested using two distinct assays, EEG and GCaMP, to see if there were discrepancies between the results found in the behavioral assays and those observed in the EEG and/or GCaMP assays. Three of the four anti-seizure drugs/compounds reduced PTZ-induced seizures in all the three assay types. Overall, there was no significant difference between pretreating and acute treatment of anti-seizure drugs in the behavioral models; thus only acute administration was used in the EEG and GCaMP assays. Future experiments could be used to determine whether pretreatment in EEG and GCaMP assays shows any difference with behavior. These assays corroborated the effectiveness of the behavioral assays’ ability to determine anti-seizure drug efficacy.

In this study our aim was to use the optimized assay to assess the efficacy of four different anti-seizure drugs/compounds and validate our behavioral assessments using two additional activity measures. CBZ was previously found to be ineffective in reducing seizure behavior and EEG activity in the zebrafish PTZ model (Bariban et al., 2005; Afrikanova et al., 2013), which contrasts with our findings. However, we used a lower concentration of PTZ over a longer time period. Interestingly, Gupta et al. (2014) found that CBZ reduced PTZ induced seizure behavior in adult zebrafish. LTG was previously shown to be ineffective in reducing seizure behavior and EEG activity in the zebrafish PTZ model (Afrikanova et al., 2013). Our behavior results were consistent with these findings, but our EEG and GCaMP studies showed LTG reduced neural activity in the zebrafish PTZ model. TPR was previously shown to be effective in reducing seizure behavior and ineffective in EEG activity in the zebrafish PTZ model (Afrikanova et al., 2013). Our results showed TPR reduced behavior and neural activity in the zebrafish PTZ model. It is worth noting that while LTG did not reduce seizure activity in our behavioral model. LTG and TPR were shown to have an anticonvulsant effect in an adult zebrafish behavioral (Pierog et al., 2021).

A candidate anti-seizure compounds, GS967, that has shown efficacy in preclinical models (Anderson et al., 2014; Baker et al., 2018) showed the greatest attenuation of PTZ induced seizure like behavior, and this compound reduced EEG and GCaMP activity, thus reducing PTZ-induced seizures in all the three assay types. We were able to clearly determine the effectiveness these anti-seizure drugs have on seizures by comparing the drugs over different assays, which helped resolve variation seen in the literature. The behavioral assay allows higher throughput evaluation of candidate compounds, and the EEG and GCAMP assays can validate potential anti-seizure compounds.

## Data Availability

The raw data supporting the conclusion of this article will be made available by the authors, without undue reservation.
