# Supplementary material for: Neural Activity Correlates With Behavior Effects of Anti-Seizure Drugs Efficacy Using the Zebrafish Pentylenetetrazol Seizure Model
Source: Front Pharmacol. 2022 Apr 12;13:836573. doi: 10.3389/fphar.2022.836573 (PMC9041662; doi:10.3389/fphar.2022.836573)
Supplement: Supplementary file 1 [file Table5.pdf]

Supplemental Data
Milder, Zybura, Cummins and Marrs
Neural Activity Correlates with Behavior Effects of Anti-Seizure Drugs Efficacy Using the Zebrafish Pentylenetetrazol Seizure Model

Table S5. Statistical Results for behavioral profile of zebrafish larvae exposed to GS967
The table displays the results of both the two-way ANOVA (Fig 5A) and one-way ANOVA (Fig 5B) performed for Figures 5 (A and B) and the resulting post-hoc Tukey comparisons and accompanying p-values for groups marked for significance within the Figure 5 graphs.

Table S5: Statistical Results for GS967 Assay

	**Two-way ANOVA table results**		
	**DF**	**F (DFn, DFd)**	**P value**
**Time x Column Factor**	30	F (30, 2065) = 3.372	P<0.0001
**Time**	5	F (3.665, 1514) = 1.920	P=0.1109
**Column Factor**	6	F (6, 413) = 48.42	P<0.0001
**Subject**	413	F (413, 2065) = 4.931	P<0.0001
**Residual**	2065		
	**Two-way ANOVA Tukey's Post-hoc Results**		
**Tukey's multiple comparisons test**	**Adjusted P Value**	**Tukey's multiple comparisons test**	**Adjusted P Value**
**Time period: 0 -15 minutes**		**Time period: 45 - 60 minutes**	
EM vs. 0.1 % DMSO	0.4063	EM vs. 0.1 % DMSO	>0.9999
EM vs. 10 mM PTZ	0.0298	EM vs. 10 mM PTZ	<0.0001
EM vs. 0.05 µm GS967 - pretreatment	0.0016	EM vs. 0.05 µm GS967 - pretreatment	0.003
EM vs. 0.05 µm GS967 acute	0.3709	EM vs. 0.05 µm GS967 acute	0.0572
EM vs. 0.05 µm GS967 - pretreatment	0.9989	EM vs. 0.05 µm GS967 - pretreatment	0.638
EM vs. 10 mM PTZ / 0.05 µm GS967 acute	0.0192	EM vs. 10 mM PTZ / 0.05 µm GS967 acute	0.9644
10 mM PTZ vs. 10 mM PTZ / 0.05 µm GS967 - pretreatment	0.0007	10 mM PTZ vs. 10 mM PTZ / 0.05 µm GS967 - pretreatment	0.001
10 mM PTZ vs. 10 mM PTZ / 0.05 µM GS967 acute	<0.0001	10 mM PTZ vs. 10 mM PTZ / 0.05 µM GS967 acute	<0.0001
**Time period: 15 - 30 minutes**		**Time period: 60 - 75 minutes**	
EM vs. 0.1 % DMSO	0.995	EM vs. 0.1 % DMSO	0.9993
EM vs. 10 mM PTZ	0.031	EM vs. 10 mM PTZ	<0.0001
EM vs. 0.05 µm GS967 - pretreatment	0.0077	EM vs. 0.05 µm GS967 - pretreatment	0.0999
EM vs. 0.05 µm GS967 acute	0.0196	EM vs. 0.05 µm GS967 acute	0.0323
EM vs. 0.05 µm GS967 - pretreatment	0.9275	EM vs. 0.05 µm GS967 - pretreatment	0.0933
EM vs. 10 mM PTZ / 0.05 µm GS967 acute	0.9614	EM vs. 10 mM PTZ / 0.05 µm GS967 acute	0.0556
10 mM PTZ vs. 10 mM PTZ / 0.05 µm GS967 - pretreatment	0.2035	10 mM PTZ vs. 10 mM PTZ / 0.05 µm GS967 - pretreatment	0.0002
10 mM PTZ vs. 10 mM PTZ / 0.05 µM GS967 acute	0.0006	10 mM PTZ vs. 10 mM PTZ / 0.05 µM GS967 acute	<0.0001
**Time period: 30 - 45 minutes**		**Time period: 75 - 90 minutes**	
EM vs. 0.1 % DMSO	>0.9999	EM vs. 0.1 % DMSO	0.9999
EM vs. 10 mM PTZ	<0.0001	EM vs. 10 mM PTZ	<0.0001
EM vs. 0.05 µm GS967 - pretreatment	0.0029	EM vs. 0.05 µm GS967 - pretreatment	0.0025
EM vs. 0.05 µm GS967 acute	0.0031	EM vs. 0.05 µm GS967 acute	0.0537
EM vs. 0.05 µm GS967 - pretreatment	0.3629	EM vs. 0.05 µm GS967 - pretreatment	0.0246
EM vs. 10 mM PTZ / 0.05 µm GS967 acute	0.9993	EM vs. 10 mM PTZ / 0.05 µm GS967 acute	0.0161
10 mM PTZ vs. 10 mM PTZ / 0.05 µm GS967 - pretreatment	0.0162	10 mM PTZ vs. 10 mM PTZ / 0.05 µm GS967 - pretreatment	0.0008
10 mM PTZ vs. 10 mM PTZ / 0.05 µM GS967 acute	<0.0001	10 mM PTZ vs. 10 mM PTZ / 0.05 µM GS967 acute	0.0002
	**One-way ANOVA table results**		
	**DF**	**F (DFn, DFd)**	**P value**
**Treatment (between columns)**	6	**F (DFn, DFd)**	**P value**
**Residual (within columns)**	413	F (6, 413) = 48.42	P<0.0001
**Total**	419		
	**One-way ANOVA Tukey's Post-hoc Results**		
**Tukey's multiple comparisons test**	**Adjusted P Value**		
EM vs. 0.1 % DMSO	0.9955		
EM vs. 10 mM PTZ	<0.0001		
EM vs. 0.05 µm GS967 - pretreatment	0.0025		
EM vs. 0.05 µm GS967 acute	0.0234		
EM vs. 0.05 µm GS967 - pretreatment	0.2294		
EM vs. 10 mM PTZ / 0.05 µm GS967 acute	>0.9999		
10 mM PTZ vs. 10 mM PTZ / 0.05 µm GS967 - pretreatment	<0.0001		
10 mM PTZ vs. 10 mM PTZ / 0.05 µM GS967 acute	<0.0001		
