# Supplementary material for: Neural Activity Correlates With Behavior Effects of Anti-Seizure Drugs Efficacy Using the Zebrafish Pentylenetetrazol Seizure Model
Source: Front Pharmacol. 2022 Apr 12;13:836573. doi: 10.3389/fphar.2022.836573 (PMC9041662; doi:10.3389/fphar.2022.836573)
Supplement: Supplementary file 2 [file Table4.pdf]

Supplemental Data
Milder, Zybura, Cummins and Marrs
Neural Activity Correlates with Behavior Effects of Anti-Seizure Drugs Efficacy Using the Zebrafish Pentylenetetrazol Seizure Model

Table S4. Statistical Results for behavioral profile of zebrafish larvae exposed to topiramate
The table displays the results of both the two-way ANOVA (Fig 4A) and one-way ANOVA (Fig 4B) performed for Figures 4 (A and B) and the resulting post-hoc Tukey comparisons and accompanying p-values for groups marked for significance within the Figure 4 graphs.

Table S4: Statistical Results for TPR Assay

	**Two-way ANOVA table results**		
	**DF**	**F (DFn, DFd)**	**P value**
**Time x Column Factor**	30	F (30, 3150) = 6.747	P<0.0001
**Time**	5	F (3.798, 2393) = 16.45	P<0.0001
**Column Factor**	6	F (6, 630) = 86.77	P<0.0001
**Subject**	630	F (630, 3150) = 2.710	P<0.0001
**Residual**	3150		
	**Two-way ANOVA Tukey's Post-hoc Results**		
**Tukey's multiple comparisons test**	**Adjusted P Value**	**Tukey's multiple comparisons test**	**Adjusted P Value**
**Time period: 0 -15 minutes**		**Time period: 45 - 60 minutes**	
EM vs. 0.1 % DMSO	0.9846	EM vs. 0.1 % DMSO	0.9978
EM vs. 10 mM PTZ	0.0052	EM vs. 10 mM PTZ	<0.0001
EM vs. 200 µm TPR - pretreatment	0.0121	EM vs. 200 µm TPR - pretreatment	0.9862
EM vs. 200 µm TPR acute	0.9901	EM vs. 200 µm TPR acute	0.0408
EM vs. 200 µm TPR - pretreatment	>0.9999	EM vs. 200 µm TPR - pretreatment	<0.0001
EM vs. 10 mM PTZ / 200 µm TPR acute	0.0022	EM vs. 10 mM PTZ / 200 µm TPR acute	<0.0001
10 mM PTZ vs. 10 mM PTZ / 200 µm TPR - pretreatment	0.033	10 mM PTZ vs. 10 mM PTZ / 200 µm TPR - pretreatment	0.6469
10 mM PTZ vs. 10 mM PTZ / 200 µM TPR acute	<0.0001	10 mM PTZ vs. 10 mM PTZ / 200 µM TPR acute	0.0014
**Time period: 15 - 30 minutes**		**Time period: 60 - 75 minutes**	
EM vs. 0.1 % DMSO	0.5761	EM vs. 0.1 % DMSO	0.7181
EM vs. 10 mM PTZ	<0.0001	EM vs. 10 mM PTZ	<0.0001
EM vs. 200 µm TPR - pretreatment	0.7773	EM vs. 200 µm TPR - pretreatment	>0.9999
EM vs. 200 µm TPR acute	0.0256	EM vs. 200 µm TPR acute	0.1545
EM vs. 200 µm TPR - pretreatment	<0.0001	EM vs. 200 µm TPR - pretreatment	<0.0001
EM vs. 10 mM PTZ / 200 µm TPR acute	<0.0001	EM vs. 10 mM PTZ / 200 µm TPR acute	0.0007
10 mM PTZ vs. 10 mM PTZ / 200 µm TPR - pretreatment	0.3473	10 mM PTZ vs. 10 mM PTZ / 200 µm TPR - pretreatment	0.8316
10 mM PTZ vs. 10 mM PTZ / 200 µM TPR acute	0.285	10 mM PTZ vs. 10 mM PTZ / 200 µM TPR acute	0.0002
**Time period: 30 - 45 minutes**		**Time period: 75 - 90 minutes**	
EM vs. 0.1 % DMSO	0.9995	EM vs. 0.1 % DMSO	>0.9999
EM vs. 10 mM PTZ	<0.0001	EM vs. 10 mM PTZ	<0.0001
EM vs. 200 µm TPR - pretreatment	0.7738	EM vs. 200 µm TPR - pretreatment	0.8247
EM vs. 200 µm TPR acute	0.0171	EM vs. 200 µm TPR acute	0.001
EM vs. 200 µm TPR - pretreatment	<0.0001	EM vs. 200 µm TPR - pretreatment	<0.0001
EM vs. 10 mM PTZ / 200 µm TPR acute	<0.0001	EM vs. 10 mM PTZ / 200 µm TPR acute	0.5344
10 mM PTZ vs. 10 mM PTZ / 200 µm TPR - pretreatment	0.3762	10 mM PTZ vs. 10 mM PTZ / 200 µm TPR - pretreatment	0.0871
10 mM PTZ vs. 10 mM PTZ / 200 µM TPR acute	0.0148	10 mM PTZ vs. 10 mM PTZ / 200 µM TPR acute	0.0002
	**One-way ANOVA table results**		
	**DF**	**F (DFn, DFd)**	**P value**
**Treatment (between columns)**	6	**F (DFn, DFd)**	**P value**
**Residual (within columns)**	35	F (6, 35) = 28.91	P<0.0001
**Total**	41		

	**One-way ANOVA Tukey's Post-hoc Results**
**Tukey's multiple comparisons test**	**Adjusted P Value**
EM vs. 0.1 % DMSO	0.998
EM vs. 10 mM PTZ	<0.0001
EM vs. 200 µm TPR - pretreatment	0.9749
EM vs. 200 µm TPR acute	0.9682
EM vs. 200 µm TPR - pretreatment	<0.0001
EM vs. 10 mM PTZ / 200 µm TPR acute	0.002
10 mM PTZ vs. 10 mM PTZ / 200 µm TPR - pretreatment	0.0407
10 mM PTZ vs. 10 mM PTZ / 200 µM TPR acute	0.0013
