# Supplementary material for: Neural Activity Correlates With Behavior Effects of Anti-Seizure Drugs Efficacy Using the Zebrafish Pentylenetetrazol Seizure Model
Source: Front Pharmacol. 2022 Apr 12;13:836573. doi: 10.3389/fphar.2022.836573 (PMC9041662; doi:10.3389/fphar.2022.836573)
Supplement: Supplementary file 3 [file Table1.pdf]

Supplemental Data
Milder, Zybura, Cummins and Marrs
Neural Activity Correlates with Behavior Effects of Anti-Seizure Drugs Efficacy Using the Zebrafish Pentylenetetrazol Seizure Model

Table S1. Statistical Results for behavioral profile of zebrafish larvae exposed to PTZ

The table displays the results of both the two-way ANOVA (Fig 1A) and one-way ANOVA (Fig 1B) performed for Figures 1 (A and B) and the resulting post-hoc Tukey comparisons and accompanying p-values for groups marked for significance within the Figure 1 graphs.

Table S1: Statistical Results for PTZ Assay

	Two-way ANOVA table results		
	**DF**	**F (DFn, DFd)**	**P value**
**Time x Column Factor**	20	F (20, 1725) = 14.82	P<0.0001
**Time**	5	F (3.149, 1086) = 47.64	P<0.0001
**Column Factor**	4	F (4, 345) = 58.51	P<0.0001
**Subject**	345	F (345, 1725) = 3.008	P<0.0001
**Residual**	1725		
	**Two-way ANOVA Tukey's Post-hoc Results**		
**Tukey's multiple comparisons test**	**Adjusted P Value**	**Tukey's multiple comparisons test**	**Adjusted P Value**
**Time period: 0 -15 minutes**		**Time period: 45 - 60 minutes**	
EM vs. 5 mM PTZ	0.9161	EM vs. 5 mM PTZ	0.0027
EM vs. 10 mM PTZ	<0.0001	EM vs. 10 mM PTZ	<0.0001
EM vs. 20 mM PTZ	<0.0001	EM vs. 20 mM PTZ	0.67
EM vs. 40 mM PTZ	<0.0001	EM vs. 40 mM PTZ	0.1085
**Time period: 15 - 30 minutes**		**Time period: 60 - 75 minutes**	
EM vs. 5 mM PTZ	0.0133	EM vs. 5 mM PTZ	0.0157
EM vs. 10 mM PTZ	<0.0001	EM vs. 10 mM PTZ	<0.0001
EM vs. 20 mM PTZ	<0.0001	EM vs. 20 mM PTZ	0.9048
EM vs. 40 mM PTZ	0.0101	EM vs. 40 mM PTZ	0.0007
**Time period: 30 - 45 minutes**		**Time period: 75 - 90 minutes**	
EM vs. 5 mM PTZ	0.0932	EM vs. 5 mM PTZ	0.0778
EM vs. 10 mM PTZ	<0.0001	EM vs. 10 mM PTZ	<0.0001
EM vs. 20 mM PTZ	0.1367	EM vs. 20 mM PTZ	0.2259
	**One-way ANOVA table results**		
	**DF**	**F (DFn, DFd)**	**P value**
**Treatment (between columns)**	4	F (4, 220) = 34.91	P<0.0001
**Residual (within columns)**	220		
**Total**	224		
	**One-way ANOVA Tukey's Post-hoc Results**		
**Tukey's multiple comparisons test**	**Adjusted P Value**		
EM vs. 5 mM PTZ	0.3342		
EM vs. 10 mM PTZ	<0.0001		
EM vs. 20 mM PTZ	<0.0001		
EM vs. 40 mM PTZ	0.9133		
