# Supplementary material for: Neural Activity Correlates With Behavior Effects of Anti-Seizure Drugs Efficacy Using the Zebrafish Pentylenetetrazol Seizure Model
Source: Front Pharmacol. 2022 Apr 12;13:836573. doi: 10.3389/fphar.2022.836573 (PMC9041662; doi:10.3389/fphar.2022.836573)
Supplement: Supplementary file 4 [file Table2.pdf]

Supplemental Data
Milder, Zybura, Cummins and Marrs
Neural Activity Correlates with Behavior Effects of Anti-Seizure Drugs Efficacy Using the Zebrafish Pentylenetetrazol Seizure Model

Table S2. Statistical Results for behavioral profile of zebrafish larvae exposed to carbamazepine

The table displays the results of both the two-way ANOVA (Fig 2A) and one-way ANOVA (Fig 2B) performed for Figures 2 (A and B) and the resulting post-hoc Tukey comparisons and accompanying p-values for groups marked for significance within the Figure 2 graphs.

Table S2: Statistical Results for CBZ Assay

	**Two-way ANOVA table results**		
	**DF**	**F (DFn, DFd)**	**P value**
**Time x Column Factor**	30	F (30, 2625) = 5.171	P<0.0001
**Time**	5	F (3.761, 1975) = 14.03	P<0.0001
**Column Factor**	6	F (6, 525) = 51.76	P<0.0001
**Subject**	525	F (525, 2625) = 3.701	P<0.0001
**Residual**	2625		
	**Two-way ANOVA Tukey's Post-hoc Results**		
**Tukey's multiple comparisons test**	**Adjusted P Value**	**Tukey's multiple comparisons test**	**Adjusted P Value**
**Time period: 0 -15 minutes**		**Time period: 45 - 60 minutes**	
EM vs. 0.1 % DMSO	>0.9999	EM vs. 0.1 % DMSO	>0.9999
EM vs. 10 mM PTZ	0.0263	EM vs. 10 mM PTZ	<0.0001
EM vs. 100 µM CBZ - pretreatment	<0.0001	EM vs. 100 µM CBZ - pretreatment	0.0109
EM vs. 100 µM CBZ acute	0.0022	EM vs. 100 µM CBZ acute	<0.0001
EM vs. 10 mM PTZ / 100 µM CBZ - pretreatment	0.1683	EM vs. 10 mM PTZ / 100 µM CBZ - pretreatment	0.1259
EM vs. 10 mM PTZ / 100 µM CBZ acute	0.4155	EM vs. 10 mM PTZ / 100 µM CBZ acute	0.0014
10 mM PTZ vs. 10 mM PTZ / 100 µM CBZ - pretreatment	0.9963	10 mM PTZ vs. 10 mM PTZ / 100 µM CBZ - pretreatment	<0.0001
10 mM PTZ vs. 10 mM PTZ / 100 µM CBZ acute	0.8847	10 mM PTZ vs. 10 mM PTZ / 100 µM CBZ acute	0.0023
**Time period: 15 - 30 minutes**		**Time period: 60 - 75 minutes**	
EM vs. 0.1 % DMSO	0.9996	EM vs. 0.1 % DMSO	0.9628
EM vs. 10 mM PTZ	0.0036	EM vs. 10 mM PTZ	<0.0001
EM vs. 100 µM CBZ - pretreatment	<0.0001	EM vs. 100 µM CBZ - pretreatment	0.0016
EM vs. 100 µM CBZ acute	<0.0001	EM vs. 100 µM CBZ acute	<0.0001
EM vs. 10 mM PTZ / 100 µM CBZ - pretreatment	0.0136	EM vs. Group F	0.3976
EM vs. 10 mM PTZ / 100 µM CBZ acute	0.9278	EM vs. 10 mM PTZ / 100 µM CBZ acute	0.001
10 mM PTZ vs. 10 mM PTZ / 100 µM CBZ - pretreatment	0.4954	10 mM PTZ vs. Group F	0.0098
10 mM PTZ vs. 10 mM PTZ / 100 µM CBZ acute	0.0576	10 mM PTZ vs. 10 mM PTZ / 100 µM CBZ acute	0.4587
**Time period: 30 - 45 minutes**		**Time period: 75 - 90 minutes**	
EM vs. 0.1 % DMSO	0.8612	EM vs. 0.1 % DMSO	>0.9999
EM vs. 10 mM PTZ	<0.0001	EM vs. 10 mM PTZ	<0.0001
EM vs. 100 µM CBZ - pretreatment	<0.0001	EM vs. 100 µM CBZ - pretreatment	<0.0001
EM vs. 100 µM CBZ acute	<0.0001	EM vs. 100 µM CBZ acute	<0.0001
EM vs. 10 mM PTZ / 100 µM CBZ - pretreatment	0.0828	EM vs. Group F	0.413
EM vs. 10 mM PTZ / 100 µM CBZ acute	0.0159	EM vs. 10 mM PTZ / 100 µM CBZ acute	0.0015
10 mM PTZ vs. 10 mM PTZ / 100 µM CBZ - pretreatment	0.0003	10 mM PTZ vs. Group F	0.203
10 mM PTZ vs. 10 mM PTZ / 100 µM CBZ acute	0.0027	10 mM PTZ vs. 10 mM PTZ / 100 µM CBZ acute	0.9853
	**One-way ANOVA table results**		
	**DF**	**F (DFn, DFd)**	**P value**
**Treatment (between columns)**	DF	**F (DFn, DFd)**	**P value**
**Residual (within columns)**	6	F (6, 525) = 51.76	P<0.0001
**Total**	525		
	**One-way ANOVA Tukey's Post-hoc Results**		
**Tukey's multiple comparisons test**	**Adjusted P Value**		
EM vs. 0.1 % DMSO	>0.9999		
EM vs. 10 mM PTZ	<0.0001		
EM vs. 100 µM CBZ - pretreatment	0.0031		
EM vs. 100 µM CBZ acute	0.0005		
EM vs. 10 mM PTZ / 100 µM CBZ - pretreatment	0.0155		
EM vs. 10 mM PTZ / 100 µM CBZ acute	<0.0001		
10 mM PTZ vs. 10 mM PTZ / 100 µM CBZ - pretreatment	<0.0001		
10 mM PTZ vs. 10 mM PTZ / 100 µM CBZ acute	<0.0001		
