# Supplementary material for: Neural Activity Correlates With Behavior Effects of Anti-Seizure Drugs Efficacy Using the Zebrafish Pentylenetetrazol Seizure Model
Source: Front Pharmacol. 2022 Apr 12;13:836573. doi: 10.3389/fphar.2022.836573 (PMC9041662; doi:10.3389/fphar.2022.836573)
Supplement: Supplementary file 5 [file Table6.pdf]

Supplemental Data
Milder, Zybura, Cummins and Marrs
Neural Activity Correlates with Behavior Effects of Anti-Seizure Drugs Efficacy Using the Zebrafish Pentylenetetrazol Seizure Model

Table S6. Statistical Results for Relative Midbrain GFP Activity

The table displays the results of the one-way ANOVA (Fig 6B) performed for Figure 6B and the resulting post-hoc Tukey comparisons and accompanying p-values for groups marked for significance within the Figure 6 graph.

Table S6: Statistical Results for Relative Midbrain GFP Intensity

	One-way ANOVA table results		
	**DF**	**F (DFn, DFd)**	**P value**
**Treatment (between columns)**	7	F (7, 88) = 36.70	P<0.0001
**Residual (within columns)**	88		
**Total**	95		

	One-way ANOVA Tukey's Post-hoc Results
**Tukey's multiple comparisons test**	**Adjusted P Value**
EM vs. 0.1 % DMSO	0.9932
EM vs. 10 mM PTZ	<0.0001
EM vs. 10 mM PTZ / 100 µM CBZ	<0.0001
EM vs. 10 mM PTZ / 200 µM TPR	<0.0001
EM vs. 10 mM PTZ / 100 µM LTG	<0.0001
EM vs. 10 mM PTZ / 0.05 µM GS967	0.9995
EM vs. EM-15	>0.9999
