# Supplementary material for: Neural Activity Correlates With Behavior Effects of Anti-Seizure Drugs Efficacy Using the Zebrafish Pentylenetetrazol Seizure Model
Source: Front Pharmacol. 2022 Apr 12;13:836573. doi: 10.3389/fphar.2022.836573 (PMC9041662; doi:10.3389/fphar.2022.836573)
Supplement: Supplementary file 6 [file Table3.pdf]

Supplemental Data
Milder, Zybura, Cummins and Marrs
Neural Activity Correlates with Behavior Effects of Anti-Seizure Drugs Efficacy Using the Zebrafish Pentylenetetrazol Seizure Model

Table S3. Statistical Results for behavioral profile of zebrafish larvae exposed to lamotrigine
The table displays the results of both the two-way ANOVA (Fig 3A) and one-way ANOVA (Fig 3B) performed for Figures 3 (A and B) and the resulting post-hoc Tukey comparisons and accompanying p-values for groups marked for significance within the Figure 3 graphs.

Table S3: Statistical Results for LTG Assay

	Two-way ANOVA table results		
	**DF**	**F (DFn, DFd)**	**P value**
**Time x Column Factor**	30	F (30, 2625) = 8.983	P<0.0001
**Time**	5	F (2.762, 1450) = 0.7716	P=0.5002
**Column Factor**	6	F (6, 525) = 29.19	P<0.0001
**Subject**	525	F (525, 2625) = 5.437	P<0.0001
**Residual**	2625		
	**Two-way ANOVA Tukey's Post-hoc Results**		
**Tukey's multiple comparisons test**	**Adjusted P Value**	**Tukey's multiple comparisons test**	**Adjusted P Value**
**Time period: 0 -15 minutes**		**Time period: 45 - 60 minutes**	
EM vs. 0.1 % DMSO	0.863	EM vs. 0.1 % DMSO	0.9335
EM vs. 10 mM PTZ	0.0138	EM vs. 10 mM PTZ	<0.0001
EM vs. 100 µm LTG - pretreatment	>0.9999	EM vs. 100 µm LTG - pretreatment	0.9886
EM vs. 100 µm LTG acute	0.5665	EM vs. 100 µm LTG acute	>0.9999
EM vs. 100 µm LTG - pretreatment	0.6537	EM vs. 100 µm LTG - pretreatment	0.0001
EM vs. 10 mM PTZ / 100 µm LTG acute	0.0325	EM vs. 10 mM PTZ / 100 µm LTG acute	0.0006
10 mM PTZ vs. 10 mM PTZ / 100 µm LTG - pretreatment	0.2824	10 mM PTZ vs. 10 mM PTZ / 100 µm LTG - pretreatment	0.1745
10 mM PTZ vs. 10 mM PTZ / 100 µM LTG acute	>0.9999	10 mM PTZ vs. 10 mM PTZ / 100 µM LTG acute	0.7238
**Time period: 15 - 30 minutes**		**Time period: 60 - 75 minutes**	
EM vs. 0.1 % DMSO	0.8197	EM vs. 0.1 % DMSO	0.945
EM vs. 10 mM PTZ	0.0007	EM vs. 10 mM PTZ	<0.0001
EM vs. 100 µm LTG - pretreatment	0.7785	EM vs. 100 µm LTG - pretreatment	0.9127
EM vs. 100 µm LTG acute	0.9935	EM vs. 100 µm LTG acute	>0.9999
EM vs. 100 µm LTG - pretreatment	<0.0001	EM vs. 100 µm LTG - pretreatment	0.0026
EM vs. 10 mM PTZ / 100 µm LTG acute	0.0741	EM vs. 10 mM PTZ / 100 µm LTG acute	0.0054
10 mM PTZ vs. 10 mM PTZ / 100 µm LTG - pretreatment	0.148	10 mM PTZ vs. 10 mM PTZ / 100 µm LTG - pretreatment	<0.0001
10 mM PTZ vs. 10 mM PTZ / 100 µM LTG acute	0.0619	10 mM PTZ vs. 10 mM PTZ / 100 µM LTG acute	>0.9999
**Time period: 30 - 45 minutes**		**Time period: 75 - 90 minutes**	
EM vs. 0.1 % DMSO	0.9983	EM vs. 0.1 % DMSO	0.993
EM vs. 10 mM PTZ	<0.0001	EM vs. 10 mM PTZ	<0.0001
EM vs. 100 µm LTG - pretreatment	0.858	EM vs. 100 µm LTG - pretreatment	0.1641
EM vs. 100 µm LTG acute	0.9999	EM vs. 100 µm LTG acute	>0.9999
EM vs. 100 µm LTG - pretreatment	<0.0001	EM vs. 100 µm LTG - pretreatment	0.1349
EM vs. 10 mM PTZ / 100 µm LTG acute	0.0002	EM vs. 10 mM PTZ / 100 µm LTG acute	0.0001
10 mM PTZ vs. 10 mM PTZ / 100 µm LTG - pretreatment	0.9989	10 mM PTZ vs. 10 mM PTZ / 100 µm LTG - pretreatment	0.0008
10 mM PTZ vs. 10 mM PTZ / 100 µM LTG acute	0.5785	10 mM PTZ vs. 10 mM PTZ / 100 µM LTG acute	0.9649
	**One-way ANOVA table results**		
	**DF**	**F (DFn, DFd)**	**P value**
**Treatment (between columns)**	DF	**F (DFn, DFd)**	**P value**
**Residual (within columns)**	6	F (6, 525) = 29.19	P<0.0001
**Total**	525		

	One-way ANOVA Tukey's Post-hoc Results
**Tukey's multiple comparisons test**	**Adjusted P Value**
EM vs. 0.1 % DMSO	0.9849
EM vs. 10 mM PTZ	<0.0001
EM vs. 100 µm LTG - pretreatment	0.9849
EM vs. 100 µm LTG acute	0.9997
EM vs. 100 µm LTG - pretreatment	<0.0001
EM vs. 10 mM PTZ / 100 µm LTG acute	<0.0001
10 mM PTZ vs. 10 mM PTZ / 100 µm LTG - pretreatment	0.0959
10 mM PTZ vs. 10 mM PTZ / 100 µM LTG acute	0.9111
