# Supplementary material for: Neural Activity Correlates With Behavior Effects of Anti-Seizure Drugs Efficacy Using the Zebrafish Pentylenetetrazol Seizure Model
Source: Front Pharmacol. 2022 Apr 12;13:836573. doi: 10.3389/fphar.2022.836573 (PMC9041662; doi:10.3389/fphar.2022.836573)
Supplement: Supplementary file 7 [file Table7.pdf]

Supplemental Data
Milder, Zybura, Cummins and Marrs
Neural Activity Correlates with Behavior Effects of Anti-Seizure Drugs Efficacy Using the Zebrafish Pentylenetetrazol Seizure Model

Table S7. Statistical Results for Relative Midbrain EEG Activity

The table displays the results of the one-way ANOVA (Fig 7B) performed for Figure 7B and the resulting post-hoc Tukey comparisons and accompanying p-values for groups marked for significance within the Figure 7 graph.

Table S7: Statistical Results for Relative Midbrain EEG Activity

	One-way ANOVA table results		
	**DF**	**F (DFn, DFd)**	**P value**
**Treatment (between columns)**	7	F (7, 56) = 10.74	P<0.0001
**Residual (within columns)**	56		
**Total**	63		

**Tukey's multiple comparisons test**	**Adjusted P Value**
EM vs. 0.1 % DMSO	0.9997
EM vs. 10 mM PTZ	<0.0001
EM vs. 10 mM PTZ / 100 µM CBZ	>0.9999
EM vs. 10 mM PTZ / 200 µM TPR	0.9657
EM vs. 10 mM PTZ / 100 µM LTG	0.9965
EM vs. 10 mM PTZ / 0.05 µM GS967	>0.9999
EM vs. EM-15	>0.9999

One-way ANOVA Tukey's Post-hoc Results
